# *bmdrc*: Python package for quantifying phenotypes from chemical exposures with benchmark dose modeling

**DOI:** 10.1371/journal.pcbi.1013337

**Published:** 2025-07-28

**Authors:** David J. Degnan, Lisa M. Bramer, Lisa Truong, Robyn L. Tanguay, Sara M. Gosline, Katrina M. Waters

**Affiliations:** 1 Earth and Biological Sciences Directorate, Pacific Northwest National Laboratory, Richland, Washington, United States of America; 2 Sinnhuber Aquatic Research Laboratory, Department of Environmental and Molecular Toxicology, Oregon State University, Corvallis, Oregon, United States of America; The University of Melbourne Faculty of Science, AUSTRALIA

## Abstract

Though chemical exposures are known to potentially have negative impacts on health, including contributing to chronic diseases such as cancer, the quantitative contribution of risk is not fully understood for every chemical. A commonly used approach to quantify levels of risk is to measure the proportion of organisms (such as a total number of zebrafish on a plate or mice in a cage) with abnormal behavioral responses or morphology at increasing concentrations of chemical exposure. A particular challenge with processing the proportional data from these assays is the appropriate estimation of chemical concentration levels that result in malformations or acute toxicity, as these values typically vary between experimental measurements. The recommended approach by the Environmental Protection Agency (EPA) is to fit benchmark dose curves with specific filters and model fitting steps, which are crucial to properly processing the proportional data. Several tools exist for the fitting of benchmark dose response curves, but none are standalone Python libraries built to process both morphological and behavioral data as proportions with all the EPA recommended filters, filter parameters, models, and model parameters. Thus, here we present the benchmark dose response curve (*bmdrc*) Python library, which was built to closely follow these EPA guidelines with helpful visualizations of filters and fitted model curves, and reports for reproducibility purposes. *bmdrc* is open-source and has demonstrated utility as a support package to an existing web portal for information on chemicals (https://srp.pnnl.gov). Our package will support any toxicology analysis where the response is a proportional value at increasing levels of a concentration of a chemical or chemical mixture.

## Introduction

Prolonged exposure to chemical toxicants may result in several serious adverse health effects, including cardiovascular diseases, nervous system damage, congenital birth defects, kidney and liver damage, and cancer, to name a few [1]. Of particular concern are chemicals like polycyclic aromatic hydrocarbons that may leech into the soil [2], water [3], and air [4], and can travel large distances to expose populations [5–8]. To mitigate damage from chemical toxicants, the United States Government enacted the Comprehensive Environmental Response, Compensation, and Liability Act (CERCLA) to identify and clean up high-risk sites of these toxicants, commonly referred to as “Superfund” sites [9]. As of 2024, there are over 1300 active Superfund sites on the National Priorities List [10]. Recent studies have demonstrated a shortened life expectancy on average for those living near Superfund sites (an estimated 24% of the US population) over those who do not [11]. Therefore, given the health risks of chemical exposures and the percentage of the population impacted by them, it is imperative to understand the potential biological effects that chemicals may have on human health. A common biological model to study chemical effects is zebrafish development assays due to their versatility and potential high-throughput capabilities (e.g., many samples at a time) [12].

**Fig 1:**
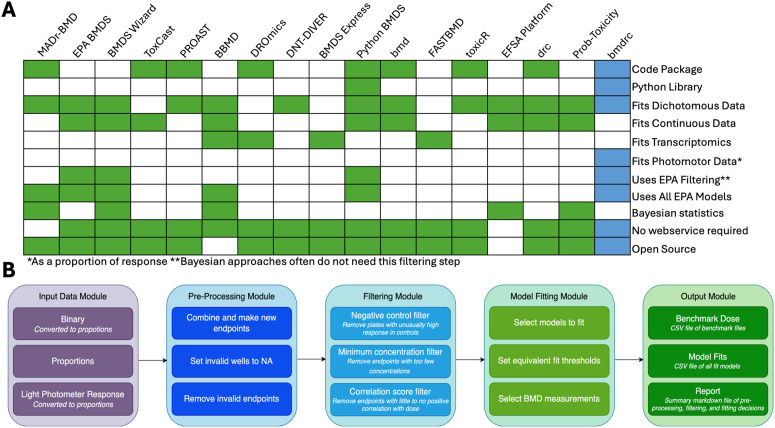
Comparison of tools with features similar to *bmdrc,* and a descriptions of the modules within the *bmdrc* package. (A) Highlighted tool features from a selection of benchmark dose modeling tools to contextualize the needs bmdrc and other existing tools fill. (B) Workflow of the *bmdrc* Python library. The large boxes are modules, which are a collection of functions with similar a purpose. The smaller boxes within the larger boxes represent individual functions within the *bmdrc* Python library.

In these studies, plates of developing fish (typically one fish per well in 296-well plates) are exposed to different concentrations of chemicals to understand the biological effects of these compounds. The response of zebrafish to chemical perturbation is generally dichotomous (also called binary): the value is a 1 if the phenotype of interest is present and a 0 if it is absent. These values are typically modeled as proportions of the number of species exhibiting the phenotype over the total number of species per sample (in this case, a plate). While these data are typically used with morphological phenotypes (e.g., physical abnormalities in response to toxicants), recent studies have demonstrated that some continuous data, like larval photomotor response (LPR) where the movement of a zebrafish larva in response to light is measured, can be transformed to dichotomous by considering a behavior normal (0) or abnormal (1) and then calculated as a proportion of abnormally behaving species per sample [12]. The Environment Protection Agency (EPA) recommended approach for quantifying phenotypes from proportional data in response to toxicant concentrations has been to fit a benchmark dose (BMD) response curve [13]. This approach is preferred to other approaches, like the No Observed Adverse Effect Level (NOAEL) and Lowest Observed Adverse Effect Level (LOAEL) methods, due to the BMD method’s increase in precision when estimating effect sizes [12,13]. Alongside this recommendation, the EPA has outlined several suggestions for fitting benchmark dose response curves to proportional data, including a maximum response in control samples, a minimum number of concentrations, and a list of preferred models, to name a few [13]. These recommendations apply to any datasets where the predictor variable is an increasing concentration and the response variable is a proportion (e.g., zebrafish per plate, mice per cage, antibody binding per plate, etc.). Researchers that wish to use their data for regulatory purposes need tools to process proportional data that also follow the EPA guidelines [14]. Though several excellent tools exist for modeling benchmark dose response curves [15–30] (Fig 1A), of the ones built specifically for dichotomous data [15–17,19,20,22,24,27–30], only a few follow all the EPA filtering recommendations [13,16,17,24]. Tools that do not follow these filters often perform model averaging or Bayesian fitting approaches instead which do not require filtering [15,17,20,25,27–30]. Of tools with filtering options, there is only one other Python library [24], which limits user options for incorporating these tools into new Python analysis pipelines. Furthermore, only a few tools offer all the EPA recommended models [15–17,24] and no existing tools convert LPR data to proportions or offer reports post-analysis that track all parameter selections, plot the amount of data removed by each filter, and offer summary statistics on the amount of data deemed eligible for modeling.

Thus, we built the benchmark dose response curve *bmdrc* Python library (Fig 1B) to closely follow the EPA recommendations for fitting dichotomous data, specifically for morphological and LPR data in a standalone format. We designed the tool for very large datasets (> 20,000 chemical and phenotype combinations) such as the data from the OSU-PNNL Superfund Portal [14]. All default parameters for filters in *bmdrc* follow the EPA guidelines so users unfamiliar with these types of data can make appropriate decisions. *bmdrc* offers several convenient features including visualizations to understand the impacts of filtering decisions, the capability to make new phenotypes from several existing ones, and the creation of report files for reproducibility purposes. Though in this work, we demonstrate the capabilities of *bmdrc* on zebrafish data, *bmdrc* has been designed to operate on any data with a proportional response (0–1 inclusive) at increasing concentrations. The *bmdrc* Python library is readily available for download at https://pypi.org/project/bmdrc/ and the raw code is available at https://github.com/PNNL-CompBio/bmdrc.

## Design and implementation

The data processing workflow of *bmdrc* can be organized into five modules (Fig 1C), which are a collection of functions with a common purpose. These modules consume three different types of data: data reported as zeroes and ones for binary (dichotomous) phenotype measurements, data reported as proportions, and data reported as continuous larval photomotor response data (LPR). Other continuous data besides LPR cannot be used with *bmdrc.* Our package has been built with test functions, example data, and tutorials called vignettes which can be accessed in the GitHub repository: https://github.com/PNNL-CompBio/bmdrc.

### Input data

For binary data, *bmdrc* requires one table that has specific columns (Table 1). The columns may have any names, as users need only specify each name of a column that holds the required information. Binary data may be in long (one measurement per line) or wide (multiple measurements per line) format. Wide format is converted to long without the need to specify a value or phenotype column, but with the assumption that the remaining columns are phenotypes. Any columns that violate this assumption should be removed if uploading the data in wide format. Proportional data is quite similar but with fewer column requirements (Table 1), as plate and well information is no longer needed if the data is a calculated proportion.

**Table 1:** A description of required columns needed to process binary (which is converted to proportions), proportional, or larval photomotor response (LPR) data in *bmdrc.*

Column Needed	Description	Data Types Required
*chemical.id*	An identifier for each chemical	Binary, proportional, LPR
*concentration*	The measured concentrations of the chemical, with units in uM	Binary, proportional, LPR
*plate.id*	An identifier for each plate	Binary, LPR
*well*	An identifier for each well	Binary, LPR
*endpoint*	The name of a phenotype to measure	Binary, proportional
*value*	(binary) A 0 or 1 indicating the absence or presence of a phenotype. (proportional) A proportion indicating the response (LPR) A measurement of movement. NA values are permitted.	Binary, proportional, LPR
*time*	The time in which the measurement was taken, where the first measurement is taken at time 0.	LPR

In the LPR class object, data is assumed to be in long format. Alongside the required columns (Table 1), users must specify the length of the light/dark exposure cycle, the time between the light and dark exposure, and whether light or dark was first. *bmdrc* will then convert the continuous data into binary data with normally (0) and abnormally (1) responding zebrafish into two phenotype measurements: a difference in the area under the curve between the dark and light cycles (called AUC) and a difference in the single movement measurement at the beginning of the dark cycle and at the end of the light cycle (called MOV), as described in full detail elsewhere [12]. These values are then converted to proportions for downstream modeling steps where NA values have been removed. For ease of use, all objects in *bmdrc* are pandas [31] DataFrames which can easily be queried by rows and columns.

### Pre-processing

After the user provides the necessary data files, the *bmdrc* package enables data cleaning through various pre-processing functions to 1) create a new phenotype from existing ones, 2) remove existing phenotype from model consideration, or 3) set wells in plates to NA to remove them from counts if well and plate information is provided. For example, if a new phenotype tracking morphological abnormalities in the head was made from the combination of cranium and jaw, users may elect to then remove the cranium and jaw phenotypes. Another pre-processing function allows for setting specific wells on plates to NA in cases where users do not want to count specific datapoints such as cases where an experimental error occurred.

### Filtering

The EPA recommends filtering steps to remove data that will have low quality fits in the downstream modeling steps [13]. The first of these filters, the negative control filter, removes plates with unusually high response in the controls (the 0 concentration measurements) if plate information is provided, with a recommended value of 50%. The second, called the minimum concentration filter, removes data with too few concentration measurements, not including the 0 concentration (negative control), with a recommended count of 3 non-zero concentration measurements. Finally, the correlation score filter removes data with a proportional response that is not positively correlated (using rank-based Spearman correlation where NAs are removed), with a recommended value of 0.2. In *bmdrc,* users may visualize the amount of data that is removed with each filtering selection with built-in plotting functions and may instead choose different parameters depending on the experiment.

### Model fitting

After data are pre-processed and filtered, users can fit all appropriate data with the “.fit_models()” function. The previous pre-processing and filtering steps are optional, though a warning message will display if models are fit with no filtering. The model fitting function follows the EPA guidelines, including the use of the seven recommended models (logistic, gamma, Weibull, log logistic, probit, log probit, multistage) and an additional model (quantal linear), as well as the recommended model selection workflow. Briefly, each model is checked first with a goodness-of-fit (GOF) test to ensure the data fits the model, where a small p-value indicates a lack of fit. Candidate models are removed if the p-value is less than 0.1. Then, the Akaike Information Criterion (AIC) metric is used to determine optimum fits, where the lowest value is indicative of the best fit. Models within 2 of the lowest AIC metric are then selected, and the lower confidence limit of the benchmark dose (BMDL) is calculated for each model. The model with the smallest BMDL is selected, as advised by the EPA [13]. All GOF, AIC, and benchmark dose measurements can be easily accessed as they are stored within the *bmdrc* objects. *bmdrc* can also visualize any curve fit to any data with the “.response_curve()” function. In cases where the treatment does not affect growth directly, a curve will not be fit to the data.

### Outputs

Users can generate detailed reports with sections detailing data input, pre-processing, filtering, and model fitting steps (see examples at https://github.com/PNNL-CompBio/bmdrc). Other outputs include several helpful summary files. The “benchmark dose” reports data with selected models, BMD10 (concentration that produces 10% of the final effect), BMDL (concentration at the lower confidence limit of the BMD10), and BMD50 (concentration that produces 50% of the final effect) measurements, if applicable. The “dose” file provides the confidence intervals for every dose and concentration measurement. Confidence intervals are calculated with the binomial distribution with the *astropy* package [32]. The “fits” file gives points along the fit curve for a distribution.

## Results

### Data description and pre-processing

To test *bmdrc* on real data, we collected the impact of 21,480 morphology and LPR datapoints for 753 chemical and chemical mixtures on Zebrafish phenotypes over a 10-year period [14]. The pre-processed data can be found on Figshare (https://figshare.com/articles/dataset/Chemical_Response_in_Zebrafish/27742668) and results post-*bmdrc* processing are visualized at https://srp.pnnl.gov. *bmdrc* was used to convert the continuous LPR data to dichotomous values, as previously described in the “Input Data” section. *bmdrc* was also used to calculate aggregate phenotypes from existing phenotypes, including a cranium phenotype from the eye, snout, and jaw phenotype; and total mortality phenotypes: one from the mortality at 24 hours and another from mortality at 5 days. *bmdrc* was also used to set values to NA for specific plates and wells with a “do not count” phentoype and the “do not count” phenotype was then removed to prevent accidentally fitting curves to it.

### Filtering and model fitting

**Fig 2:**
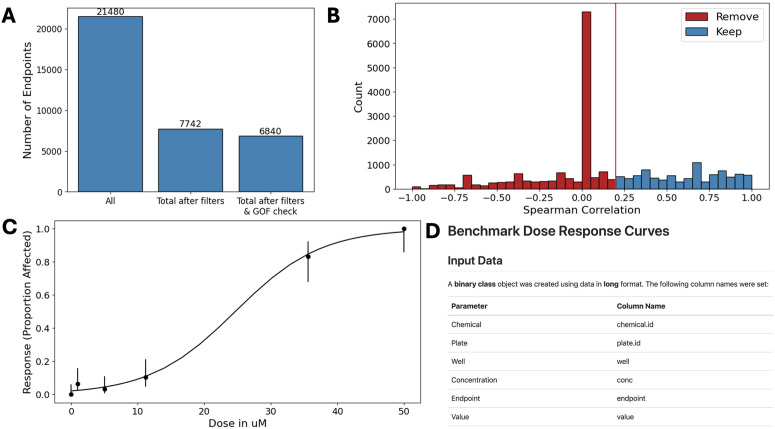
Examples of plots and reports generated by *bmdrc. * (A) A count of the total number of chemical and phenotype combinations (left), the number remaining after model eligibility filters (middle), and the number remaining after filtering and goodness-of-fit (GOF) checks (right). (B) Distribution of Spearman correlation values for every chemical and phenotype. The red bars indicate data that are not selected for downstream modeling (remove) and the blue bars indicate data that are selected (keep), based on the user-specified threshold (red line at 0.2). (C) Example of a curve generated by the *bmdrc* Python library. (D) A snippet of the first few lines of the report files which can be created in *bmdrc.*

The negative control, minimum concentration, and correlation filters were all run with the recommended EPA (default) values, as described in the filters section. 7742 phenotype and chemical combinations (~36%) passed filters (Fig 2A). The most impactful filter was the correlation filter and a helpful data visualization provided by *bmdrc* highlights the high impact (Fig 2B). An additional 902 models failed the goodness-of-fit check, for a total of 6840 (29.3%) phenotype and chemical combinations deemed acceptable for modeling (Fig 2A). From these data, most were fit to quantal linear, gamma, logistic, and log logistic models (1771, 1636, 1496, and 1412, respectively). A few were fit to the Weibull, probit, log probit, and Multistage2 models (211, 179, 132, and 3, respectively). *bmdrc* was used to visualize the generated curve fits, and an example of a logistic curve is provided (Fig 2C). Reports were generated for reproducibility purposes (Fig 2D) and can be found here: https://github.com/PNNL-CompBio/bmdrc/tree/main/example_report/publication_examples. Output files were visualized with the Superfund analytical portal (https://srp.pnnl.gov) and a command line interface (CLI) built to demonstrate how *bmdrc* was incorporated into a larger pipeline, which can be found here: https://github.com/PNNL-CompBio/srpAnalytics.

## Availability and future directions

*bmdrc* is available at: https://github.com/PNNL-CompBio/bmdrc. The tool could be expanded to several additional datatypes beyond proportional data, such as continuous data (Fig 1A). There are already several excellent implementations of these tools [16–18,20,23–26,28–30] as well as differences in requirements from the EPA for fitting these types of data due to their diverse and complex nature. *bmdrc* could wrap functionality from existing tools to diversify the data types offered and use existing studies to define filters and acceptable models for specific continuous datasets (such as fluorescence assays for enzyme activity or qt-pcr studies), instead of a “generic” pipeline for continuous data. Another future direction would be to implement model averaging and Bayesian approaches for benchmark dose calculations, for researchers interested in employing these techniques, or building a web application that would allow the use of *bmdrc* without requiring coding skills in Python*.* As new methodologies gain momentum for use in regulatory decision-making [33], tools that incorporate EPA guidelines for data filtering and benchmark dose modeling will be needed for a variety of *in vitro* assays and mixtures of continuous and dichotomous data types.
